# Bladder Injury and Urinary Tract Infection Following Low-Energy Pelvic Fracture in an Elderly Patient

**DOI:** 10.7759/cureus.76482

**Published:** 2024-12-27

**Authors:** Yuki Kataoka, Mariko Oniwa-Irie, Takuya Miyagawa

**Affiliations:** 1 Section of Clinical Epidemiology, Department of Community Medicine, Kyoto University Graduate School of Medicine, Kyoto, JPN; 2 Department of Healthcare Epidemiology, Kyoto University Graduate School of Medicine/School of Public Health, Kyoto, JPN; 3 Department of Systematic Reviewers, Scientific Research Works Peer Support Group, Osaka, JPN; 4 Department of Internal Medicine, Kyoto Min-iren Asukai Hospital, Kyoto, JPN

**Keywords:** abdominal sepsis, closed pelvic fracture, complicated urinary tract infection, fall, urinary system injuries

## Abstract

This case report presents an 86-year-old female patient who developed a urinary tract injury and infection following a pelvic fracture caused by a bedside fall during hospitalization for *Pneumocystis jirovecii* pneumonia. The patient experienced fever with chills and rigors, prompting antibiotic treatment. Imaging revealed an ischial tuberosity fracture with potential bone fragment retention in the bladder wall. Despite these complications, the patient was successfully treated with conservative management, including targeted antibiotic therapy, without surgical intervention. This case highlights the importance of considering urinary tract infections in elderly patients with pelvic fractures, even from low-energy trauma. Conservative treatment can be effective in managing extraperitoneal bladder injuries and associated infections, even in the presence of bone fragments. The successful outcome indicates that surgical intervention may not always be necessary when infection control is achieved through conservative measures.

## Introduction

Pelvic fractures following falls are becoming increasingly prevalent among elderly patients. These fractures can lead to prolonged hospitalization, decreased mobility, and mortality, especially in patients over 80 years old [1,2]. A recent case report has shown that even low-energy trauma in elderly patients can result in massive hemorrhage requiring careful management [3].

In cases of high-energy pelvic trauma, genitourinary injuries are well-documented complications that can lead to urinary tract infections, with reported rates of up to 30% [4,5]. These infections are associated with increased mortality and longer hospital stays [4]. However, there are limited studies addressing urinary tract infections following low-energy pelvic trauma, particularly in elderly patients.

Herein, we report a case of an elderly patient who developed a urinary tract injury and infection following a pelvic fracture caused by an in-hospital fall. This case demonstrates the importance of considering urinary tract infections in elderly patients with pelvic fractures, even from low-energy trauma.

## Case presentation

An 86-year-old female patient was emergently admitted with fever and respiratory failure. Prior to admission, the patient was fully independent in activities of daily living. The patient had been receiving outpatient treatment with 3 mg prednisolone per day for polymyalgia rheumatica. She was diagnosed with *Pneumocystis jirovecii* pneumonia and started treatment with sulfamethoxazole-trimethoprim. The patient became afebrile the day following admission, with C-reactive protein (CRP) levels decreasing from 7.95 mg/dL on admission to 1.92 mg/dL by post-admission day 4.

**Figure 1:**
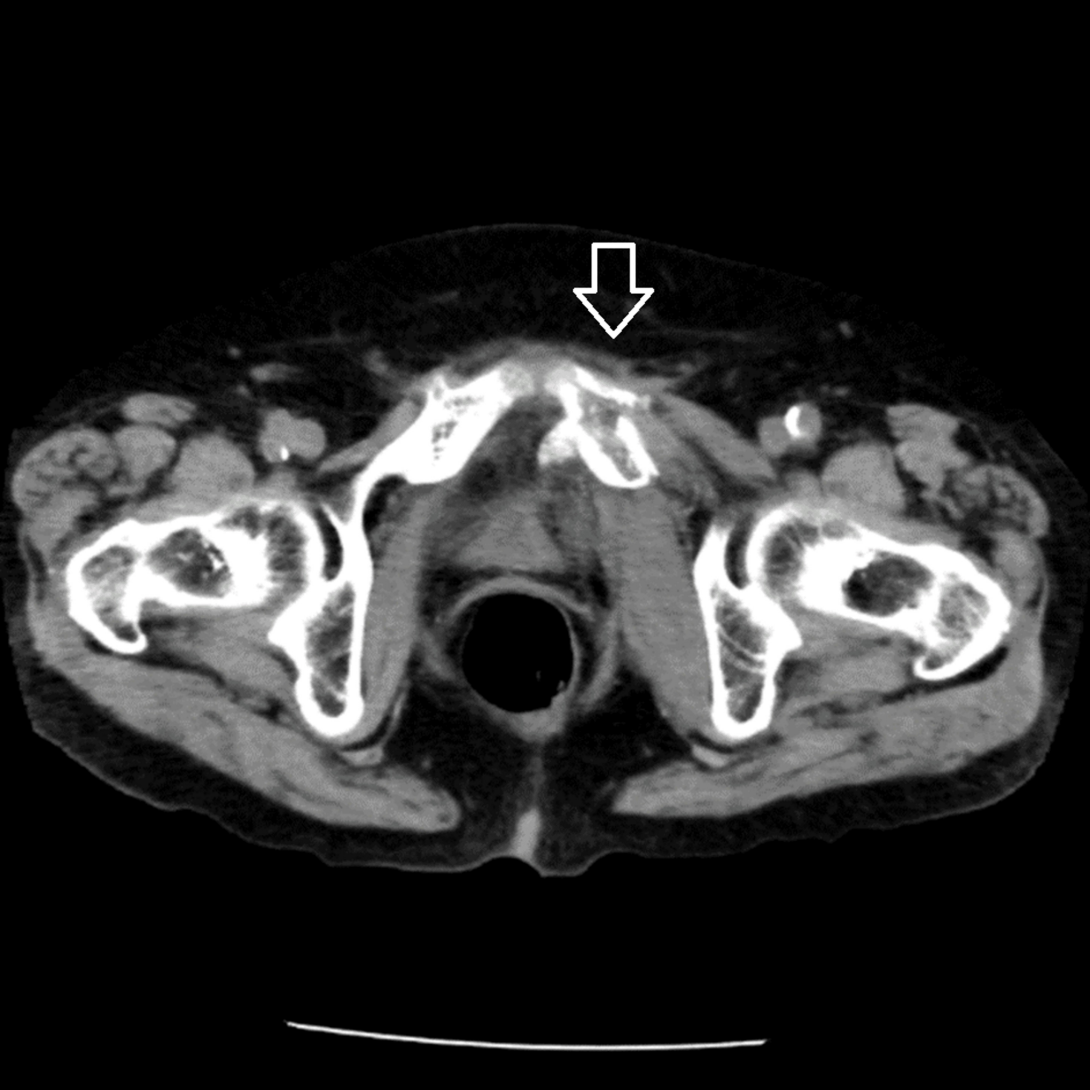
CT image immediately after the injury showing the tuberosity fracture. The arrow indicates a fracture of the left pubic bone and the resultant bone fragment.

On the 4th day of hospitalization, the patient fell and landed on her buttocks at the bedside. A computed tomography (CT) scan revealed an ischial tuberosity fracture, classified as a Tile-type A2 injury, indicating a stable pelvic ring fracture (Figure 1). Concurrent hematuria was observed, prompting urinary catheter placement for monitoring. Pre-admission urinalysis showed negative blood with 2+ bacteria. Following the injury, urinalysis revealed 3+ blood with persistent 2+ bacteria.

On post-injury day 1, hematuria resolved. On post-injury day 2, the patient developed a fever. By post-injury day 3, the clinical condition deteriorated, manifesting as fever with chills and rigors. Laboratory evaluation revealed an elevated CRP of 23.16 mg/dL. Based on these clinical and laboratory findings indicating severe systemic inflammation intravenous ceftriaxone was initiated. Although fever trends improved from the 4th day post-injury, urine culture revealed extended-spectrum beta-lactamase (ESBL) producing *Escherichia coli*. The ESBL-producing *E. coli* was found to be resistant to sulfamethoxazole-trimethoprim. Consequently, the antibiotic regimen was adjusted to cefmetazole.

**Figure 2:**
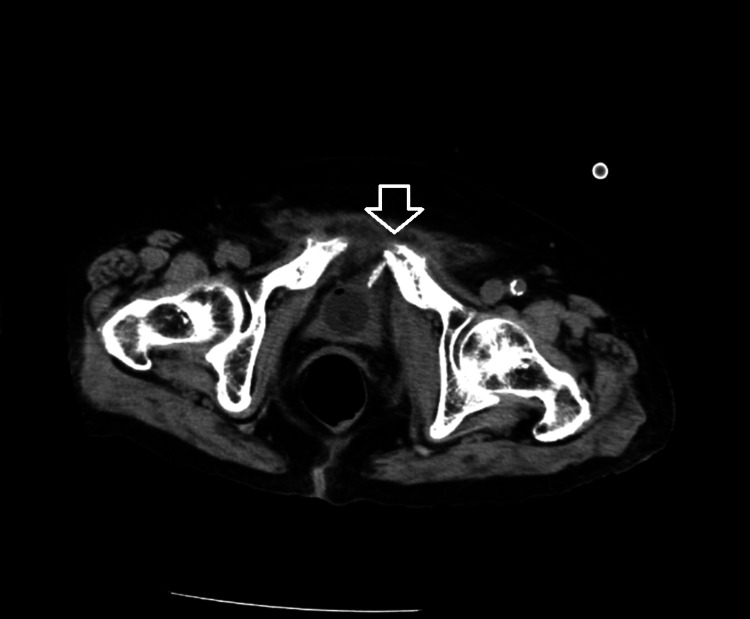
CT image taken on the 5th day post-injury. The arrow shows potential bone fragment retention in the bladder wall.

On the 5th day post-injury, the cystoscopy was performed to evaluate for visible bone fragments in the bladder, which would help determine the need for surgical intervention. The cystoscopy showed localized mucosal irregularities at the dome of the bladder but no visible bone fragments. CT imaging suggested potential bone fragment retention in the bladder wall and suspected infection at the pubic fracture site (Figure 2).

On the 36th day post-injury, the patient was transferred for potential surgical intervention. MRI findings were inconclusive for osteomyelitis or abscess. Cystoscopy, cystography, and CT scan showed no apparent leakage from the bladder. Aspiration of the fracture site yielded bloody fluid with negative culture results. Antibiotic treatment was discontinued. Following a decrease in C-reactive protein levels, the urinary catheter was removed.

On the 19th day post-transfer, the patient was readmitted to our hospital for rehabilitation. One month later, no recurrence of urinary tract infection had been observed.

## Discussion

Our case highlights three important clinical insights regarding bladder injury and urinary tract infections in elderly patients with pelvic fractures. First, while urinary system injuries are traditionally associated with high-energy trauma, even low-energy pelvic fractures in elderly patients can lead to bladder injuries due to their osteoporosis [6]. Specifically, in elderly patients, osteoporosis may contribute to complex fracture patterns even from seemingly minor trauma, increasing the risk of bladder wall injury.

We hypothesize that the bone fragment transiently penetrated the bladder wall, as evidenced by initial gross hematuria and subsequent imaging findings. The patient’s pre-existing bacteriuria (2+ on admission) combined with post-trauma clinical deterioration suggests bacterial translocation through the traumatized tissue. The concurrent bladder wall inflammation and adjacent bone involvement presented overlapping infectious foci, though definitive anatomical localization remained challenging without surgical exploration.

Second, the recognition of infection progression patterns is crucial in these cases. Although fever is a common post-fracture complication, the development of chills and rigors, as seen in our case, strongly suggests infection requiring prompt intervention [4,7]. The progression from mild symptoms to severe infection can be particularly rapid in elderly patients. Recent large-scale cohort studies have demonstrated that urinary tract infections in patients with pelvic fractures are associated with increased mortality and prolonged hospitalization [4]. Therefore, physicians should maintain a suspicion of urinary tract or osteomyelitis when fever persists in elderly patients with pelvic fractures.

Finally, this case adds to the current knowledge base regarding management strategies for bladder injuries with retained bone fragments. Although surgical intervention has been considered the standard approach for such cases [8], our experience suggests the potential effectiveness of conservative management in selected patients. This observation has particular clinical relevance for elderly patients, who often present with elevated perioperative risks [9]. The favorable outcome achieved in our case demonstrates that when adequate infection control is established through targeted antibiotic therapy and rigorous monitoring, conservative management may be a viable alternative to surgical intervention, even in cases complicated by retained bone fragments.

## Conclusions

This case shows that physicians should suspect urinary tract infections and osteomyelitis in elderly patients with pelvic fractures when fever persists, even in cases of low-energy trauma. Early recognition and appropriate intervention of these infections are crucial for optimal patient outcomes, particularly in elderly patients who may present with subtle initial symptoms that can rapidly progress to more severe conditions.

While surgical intervention is often considered standard treatment for bladder injuries with retained bone fragments, our case demonstrates that conservative treatment can be an effective option for managing urinary tract infections associated with pelvic fractures in elderly patients. This approach, combining appropriate antibiotic therapy with careful monitoring, may be particularly valuable for elderly patients who might face increased risks from surgical intervention, provided that adequate infection control is achieved through conservative measures.
